# Increasing Response Diversity to Intraverbals in Children with Autism Spectrum Disorder

**DOI:** 10.1007/s10803-019-04250-3

**Published:** 2019-10-16

**Authors:** Gabrielle T. Lee, Xiaoyi Hu, Yanhong Liu, Chouyun Zou, Xia Cheng, Qi Zhao, Jingjing Huang

**Affiliations:** 1 Faculty of Education, Western University, 1137 Western Road, Room 1105, London, ON Canada; 2 Department of Special Education, Beijing Normal University, Rm 408, YingDong Building, Xin Jie Kou Wai Da Jie #19, Beijing, China; 3 Department of Special Education, Beijing Normal University, Rm 419, YingDong Building, Xin Jie Kou Wai Da Jie #19, Beijing, China; 4 Zhuxiang School, Nong Lin Road #61, FuTian District, Shenzhen, China; 5 Faculty of Education, Education Research Center for Children with Autism, Beijing Normal University, Rm 406, YingDong Building, Xin Jie Kou Wai Da Jie #19, Beijing, China; 6 Hai Dian Modern Art Preschool, 7th Building, Luo Zhuang Xi Li, Zhi Chun Lu, HaiDian, Beijing, China

**Keywords:** Multiple control, Convergent control, Divergent control, Intraverbal behavior, Response diversity, Creativity, Autism spectrum disorder

## Abstract

The purpose of this study was to evaluate the effects of intraverbal prompts on response diversity and novelty in intraverbals posed to children with autism spectrum disorder (ASD). The intraverbal prompts involving function, feature, and class (FFC) of an item were used in the training of three questions requiring multiple responses. Two Chinese boys with ASD (aged 5–6 years) served as participants. A multiple-probe across three behaviors design was employed. The results indicated that the intraverbal prompts effectively increased the number of divergent responses to all three questions. Novel responses emerged at a low level while generalization to similar questions was not observed following the training.

The defining characteristics of autism spectrum disorder (ASD) include repetitive behavioral patterns, restricted interests or activities, and difficulties in effective social communication (American Psychiatric Association 2013). Individuals with ASD often have circumscribed interests, insist on routines, or display stereotypic behaviors. This type of deficit, conceptualized as the lack of behavioral variability, can possibly limit creativity in individuals with ASD (Neuringer 2002). From the behavioral perspective, creative behavior has been operationally defined as the diversity of responses (e.g., an increase in the number of varied responses to a question or task) and the novelty of responses (e.g., an increase in the number of new responses that have not occurred previously) (Cautilli 2004; Neto et al. 2016; Sloane et al. 1980; Winston and Baker 1985). The definition provides an in-depth understanding of certain aspects of the creative process, which can be translated into practice and guide the development of intervention and research aimed at increasing creativity in individuals with ASD.

When considered as operant behavior, creativity can be improved through verbal instructions, prompting procedures, and reinforcement. Goetz and Baer (1973) increased preschool children’s novel responses in block building simply by delivering social praise contingent upon each new pattern. Implementing the Alternative Uses Tasks in the *Torrance Tests of Creative Thinking* (TTCT; Torrance 1966), Glover and Gary (1976) designed an Unusual Uses Game for a group of fourth and fifth graders and provided instruction for the awarding of points based on the number of (a) different responses, (b) verb forms, (c) words per response, and (d) new responses. The verbal instruction, reinforcement, and repeated practice effectively increased all four outcome measures and resulted in an overall increase in the students’ TTCT scores. More recent research indicates that the lag schedules of reinforcement, which reinforce a different response from a certain number of previous ones, improved response variability to social questions for children with ASD (Lee et al. 2002; Susa and Schlinger 2012). An intraverbal training procedure has been used to increase creative play of common items in young children with ASD (Lee et al. 2019). Specifically, the children were taught to provide multiple intraverbal responses and demonstrate creative play actions using a common item (e.g., Presenting a bowl and asking, “What can you pretend with a bowl?”). Picture prompts were used to facilitate target intraverbal responses. Results indicated that the training procedure increased the number of intraverbal responses, and further, novel intraverbal responses along with play actions emerged without direct training. The above studies have targeted creative responses in various forms (i.e., play activities, written responses, and social conversation), suggesting that creative behavior is multi-faceted and can be improved through increasing the diversity or novelty of responses, or both.

The lack of response diversity or novelty in intraverbal behavior can potentially aggravate the deficiency in social communication for individuals with ASD. Intraverbal behavior, as defined by Skinner (1957), is one type of verbal behavior in which a verbal response is evoked by a preceding verbal stimulus without point-to-point correspondence between them. Conversation is an example of intraverbal behavior. Difficulties in establishing effective social communication in children with ASD is related to a lack of convergent and divergent control in intraverbal behavior (Michael et al. 2011). Convergent control in intraverbal behavior involves multiple stimuli evoking one response; divergent control involves one stimulus evoking multiple responses (Michael et al. 2011). Conditional discriminations, in which multiple relevant stimuli are dependent on each other to evoke a single response, are a type of convergent control prevalent in everyday language and tasks (Koegel et al. 2001; Sundberg and Sundberg 2011). Creative thinking tasks typically require both convergent and divergent control. For example, a similar question in TTCT asks the child to provide as many responses as possible for red things. The phrase ‘red things’ consists of two relevant stimuli (i.e., “red” and “things”), thus requiring convergent control or conditional discriminations to answer this question. That is, one must attend to both “red” and “things” to accurately respond to the task. Divergent control comes into play when the tasks require the child to provide multiple responses. The lack of convergent control or conditional discriminations may result in overselectivity (i.e., attending to one stimulus while ignoring others), which impedes skill acquisition (Koegel et al. 2001; Sundberg and Sundberg 2011). An individual with limited divergent control in intraverbal behavior, on the other hand, is likely to engage in rote responses or echolalia in social conversations (Michael et al. 2011). As overselectivity, rote responding, or echolalia are often observed in children with ASD, interventions targeting convergent control and divergent control in intraverbal behavior is fundamental to establishing effective communication.

Previous research on the intervention for complex intraverbal behavior involving both convergent and divergent control in children with ASD is limited, but the results from available studies are positive. Grannan and Rehfeldt (2012) found the emergence of multiple responses to categorical questions requiring convergent control (e.g., “Name things in the bathroom”) following the instruction in the sequence of simple tact (name an item), category tact (name the category of an item), and matching items by category for two children with ASD. After the instructional sequence, the children in the study provided a range of one to six responses without direct instruction for each categorical question. However, data on the maintenance of the derived intraverbal behavior following the intervention were not collected.

Feng et al. (2017) used picture prompts to increase the number of responses provided to categorical questions (e.g., yellow fruits, land vehicles) for a child with ASD. Although picture prompts were effective, they can potentially develop prompt dependence, as a child can still provide accurate responses by attending only to the pictures while ignoring the antecedent verbal stimulus (e.g., a categorical question) during instruction. To avoid such a problem, Lee et al. (2017) developed an intraverbal prompting procedure using the function, feature, and class (FFC) of target objects to prompt for correct responses to categorical questions. For example, an intraverbal prompt for a strawberry in the category of red things can be: “You can eat it. It has dots on it and leaves on top. It is a fruit.” This type of prompt is thematic, as the supplementary stimulus has no point-to-point correspondence between the prompt itself and the target response. Further, it presents a group of relevant stimuli requiring the exercise of convergent control to determine the target response, and therefore prompt dependency is less likely to occur. However, the categorical questions in Lee et al.’s study included only one type of categorical question (i.e., objects of five different colors). It remains unclear whether this prompting procedure is effective in improving intraverbals in other types of questions.

A review of the behavioral literature suggests that children with ASD can acquire certain aspects of creativity through systematic instructions. Learning to answer questions that require multiple responses or creative uses of common objects may provoke intraverbal responses pertaining to diversity and novelty. Establishing convergent and divergent control in intraverbal relations is essential for effective social communication. Therefore, developing and evaluating an intervention aimed at increasing response diversity while strengthening multiple controlled intraverbals is relevant (Aguirre et al. 2016; Rodriguez and Thompson 2015; Stauch et al. 2017; Wolfe et al. 2014).

In response to the call for research in multiple control of intraverbal behavior, the present study sought to extend Lee et al. (2017) by using the FFC intraverbal prompting procedure to increase response diversity to three questions modified based on TTCT. The research questions include (a) to what extent does the procedure increase the number of divergent responses provided to the target questions?, (b) to what extent does the procedure increase the number of novel responses provided to the target questions?, and (c) to what extent does the procedure increase the number of responses provided to generalization questions?

## Method

### Participants

The participants were recruited from a private inclusive preschool in Beijing, China. The preschool was located in the community and the children attended to this preschool were from middle-class families. The selection criteria included that the child had a formal diagnosis of ASD without comorbid disorders, responded to social questions, had intraverbal behavior as instructional goals in their curricular plans, and had repetitive or stereotyped speech in verbal communication as suggested by a teacher.

Dede was a 6-year-old boy diagnosed with ASD by a pediatrician using the Chinese version of the *Childhood Autism Rating Scale* (C-CARS; Lu et al. 2004; Schopler et al. 1980, 2002), the Diagnostic and Statistical Manual of Mental Disorders (DSM-5, American Psychiatric Association 2013), and the Chinese version of the Social Communication Questionnaire, Current Form (C-SCQ-C, Liu and Xu 2015; Rutter et al. 2003). His C-CARS score was 34.5, in the range of mild-to-moderate autism, and his total score of the SCQ was 16, indicating a risk for ASD. His IQ score was 83, assessed from the Chinese version of the *Wechsler Intelligence Scale for Children*-*IV* (C-WISC-IV; Wechsler 2003; Zhang 2008). Based on his assessment record, Dede’s score on the Chinese version of the Verbal Behavior Milestones Assessment and Placement Program (C-VB-MAPP; Huang and Li 2017; Sundberg 2008) was 159.5, with skills at Levels 2 and 3. Dede could use full sentences to ask for preferred items when prompted with “What do you want?” Dede could also label at least 500 common objects and receptively identify their FFC. However, he only initiated communication when requesting preferred items and did not respond to others’ requests or share items with others. He responded to questions but was often limited to rote answers (e.g., always answering “Apples and bananas” when asked, “What fruits do you like?,” “Do you like fruits?,” or “What fruits can you get in the supermarket?”). He often engaged in repetitive self-talk or delayed echolalia out of context. Dede attended a full-day inclusive kindergarten class (5–6 years old) with 30 typically developing children and two children with ASD, a headteacher, and three teaching assistants. Dede’s goals in his curricular plan included gross motor skills, verbal imitation of sentences, describing features of objects/persons/places, and pencil grasping.

Tian was a 5-year-old boy who attended the same full-day inclusive preschool as Dede but was in the prekindergarten classroom (4–5 years) with 32 typically developing children. His record indicated that his C-WISC-IV IQ score was 83. His score on the C-CARS was 32.5 in the mild-to-moderate category, and his total score of the C-SCQ-C was 15, indicating the likelihood of ASD. His C-VBMAPP score was 125.5, with all skills at Levels 2 and 3. Tian could label at least 500 items and receptively identify their FFC. Tian could use phrases to ask and answer questions. He often provided invariant responses to social questions. For example, he always answered “gummy and skittle” to questions related to food, such as “What did you eat for lunch?” or “What do you like to eat?” He also engaged in immediate and delayed echolalia during free play and did not respond or initiate social interactions with his peers. He also mixed subject pronouns, such as I, you, and he. Tian’s instructional goals in his curricular plan included playing basketball, drawing with crayons, English alphabets, and Chinese phonics.

### Setting

The study was conducted in the inclusive preschool where the participants were recruited. The preschool had seven classrooms divided by children’s age: one toddler room (2–3 years), two preschool rooms (3 years), two prekindergarten rooms (4–5 years), and two kindergarten rooms (5–6 years). Each classroom had one to three children with ASD, an intellectual disability, or other developmental delays. The children with disabilities attended regular classrooms and participated in activities with their typically developing peers in the morning and received specialized training based on their curricular plans in the afternoon. The training of the study was delivered in a one-to-one format in an individual tutoring room during recess. The follow-up sessions were conducted in each child’s home classroom in the presence of the headteacher, volunteers, and children engaging in other activities.

### Target Selection

Target questions 1 (What are red things?) and 3 (What are alternate uses for a water bottle?) were based on the questions in the TTCT. Question 2 (What are common uses for flour?) was added as a transition to Question 3. The instructors preselected 25 target answers for Question 1, 25 for Question 2, and 15 for Question 3. These answers were collected by interviewing same-aged typically developing children (all three questions) and searching the Internet (i.e., alternate uses). See Tables 1, 2, and 3 for target answers of the three target questions and their FFC used in intraverbal prompts. Two similar questions for each target question were used to test for generalization (i.e., Question 1: green, yellow things; Question 2: common uses for napkins and water; Questions 3: alternate uses for paper clips and pencils).

**Table 1:** Target answers and their FFC prompts used for red things

Target answer	FFC	Target answer	FFC
red apple	Eat, fruit, red outside yellow inside	Hot sauce	Sauce, spicy, made from chili pepper
Chinese date	Eat, dried fruit, sweet	Red bean	Eat, grain, desert soup
Tomato	Eat, fruit or vegetable, round shape	Inkpad	For stamp, office stationary, press on it
Water melon	Eat/quench thirst, fruit, green outside	Red envelope	For lucky money, rectangular, envelope
Dragon fruit	Eat, fruit, red or white inside with tiny black seeds	Couplets	For new year, decoration, on two sides of the door
Hawthorn	Eat, fruit, used for sugar-coated gourd	New year lantern	For new year, decoration, light up
Cherry	Eat, fruit, round with a core inside	Fire crackers	For new year, celebration, have noises when lit
Strawberry	Eat, fruit, small dots with green leaves on top	Fire extinguisher	Put off fire, emergency use, on building hallways
Red traffic light	Traffic sign, on street, “stop” when lit	Fire truck	Vehicle, put off fire, 119 on it
Ketch up	Food, made of tomatoes, goes with fries	Fire hydrant	Put off fire, emergency use, stick on roadside
Red rose	Flower, for bouquet, thorns on stems	Five-star flag	Represent China, has 5 stars, on the pole
Maple leaf	Turn red in autumn, size of a palm, for enjoyment or viewing	Chili pepper	Spice or vegetable, make food spicy, thin/long shape
Pomegranate	Eat, fruit, lots small red dots inside

**Table 2:** Target answers and FFC prompts for common uses of flour

Target answer	FFC	Target answer	FFC
Noodles	Eat, stripes, carbohydrate	Hamburger	Eat, meat in between buns, American
Moon cake	Eat, sweet, mid-autumn festival	Egg tart	Eat, custard on crust, dessert
Cake	Eat, dessert, birthday	Wonton	Eat, meat inside, for soup
Steamed bun	Eat, round, carbohydrate	Paste	Adhesive, sticker than glue, office stationary
Stuffed steamed bun	Eat, meat or sweet inside, snack	Grape cleaner	Cleanser, wash with water, for a fruit
Dumpling	Eat, ingot-shaped, meat inside	Play dough	Play, make anything/shape, toy
Green onion pancake	Eat, flat, green onion on top	Toast	Eat, square, breakfast or snack
Pancake	Eat, round flat with syrup on top, breakfast or snack	Oreo	Eat, white cream sandwiched chocolate crackers, snack
Chinese flat bread	Eat, breakfast, sesame seeds on top	Cup noodles	Eat, add hot water, snack
Pizza	Eat, tomato and cheese on top, Italian	Chinese fritter	Eat, breakfast, stick-shaped
Cracker	Eat, thin, snack	Donut	Eat, round with a hole in middle, dessert
Cookie	Eat, round, sweet	Chinese Gnocchi	Eat, bite-sized, for soup
Crepe	Eat, sweet or savory, lunch or snack

**Table 3:** Target answers and their FFC prompts for alternate uses of plastic water bottles

Target answer	FFC	Target answer	FFC
Flower vase	Display flowers, cylinder-shaped, table decoration	Fish tank	Keep fish, fill water, decoration
Watering can	Watering plants, can-shaped, gardening tool	Piggy bank	Keep coins, piggy shaped, save money
Pen holder	Stationary, cylinder-shaped, hold pens	Cup	Drink, hold water, cylinder-shaped
Plant/flower pot	Keep plants or flowers, soil inside, gardening tool	Stamp	Make color prints, press on paper, art tool
Kitchen canister	Store seasonings, big or small sizes, kitchenware	Bowling	Exercise, roll a ball to hit them, indoor sport
Soccer	Sport, round, kick	Funnel	Put liquid or fine grain in small opening containers, wide mouth and narrow stem, pipe
Decoration flower	Decoration, flower-shaped, art work	Thread/yarn winder	Gather thread/yarn, stick-shaped, knitting tool
Lottery box	Lottery drawing, lottery tickets inside, a box		

### Experimental Design

The study employed a multiple-probe across three behaviors design (Gast et al. 2018) to examine the functional relationship between the intraverbal prompting procedure and the acquisition of response diversity and novelty in intraverbals. The behaviors were the three target questions requiring multiple answers. The target questions were taught in the training condition and the other two similar questions were tested for generalization.

The sequence of the conditions included baseline, training, and follow-up conditions. Probe trials for target questions were conducted across all conditions. A probe trial for a target question was conducted before the training session, and the probe data were graphed and counted toward criterion. The training began with Question 1, and once the child had provided at least 10 responses to Question 1 for three consecutive probe trials, training for Question 2 was introduced. The same sequence applied to Question 3. The training condition ended when all target answers of each target question reached the mastery criterion, which required the child to provide each target answer in two consecutive probe trials. Probe trials for generalization questions were conducted in baseline and follow-up conditions.

### Response Definitions and Data Collection

The dependent variables included (a) the number of divergent responses and (b) the number of novel responses each time a question was asked in probe trials. Divergent responses refer to correct and varied multiple responses to each question (Lee et al. 2017). Correct responses were defined as the child independently provided answers relevant to the question within 3 s upon hearing the question asked. For example, in a probe trial, the child said, “red apples, fire trucks, cherries, dragon fruits, stop signs” when asked “Name red things, as many as you can.” The number of correct responses was recorded as 5 for the probe trial. Incorrect responses were defined as the child provided irrelevant answers or nonspecific answers to the question. For example, the answer “apples” was not considered a correct answer for red things, as it did not specifically refer to red ones. For the question of alternate uses, common uses for the object (e.g., using a water bottle to hold water) were not considered correct answers.

A novel response was defined as a correct response that was not introduced by the instructor or said by the child in previous trials when the question was asked (Lee et al. 2017). Following the above example, suppose the child said, “red apples, fire trucks, cherries, dragon fruits, stop signs, red grapes” in the next trial. The answer “red grapes” was not introduced by the instructor in training sessions and was absent in the child’s previous responses, so “red grapes” would be recorded as a novel response for this trial. If the child continued to provide “red grapes” in the following trials, the answer “red grapes” was counted as a correct response but not a novel one. According to this definition, correct responses emitted in baseline probe trials for the first time were considered novel responses because these answers, if any, were not taught or emitted previously.

### Procedure

#### Preference Assessment

Prior to the training, multiple-stimulus-without-replacement preference assessments were conducted based on the procedure described by DeLeon and Iwata (1996) to identify each child’s preferred items. A total of 10 potential preferred items listed by each child’s teacher were evaluated in the assessment. The top-ranked seven or eight items were used as reinforcers for probe and training sessions. Before each session, the instructor presented all preferred items to the child for him to select one item as a reinforcer for that session.

#### Pre-experimental Assessment

The two children were tested to ensure they could tact the target answers and receptively identify the associated FFC. Tacting the target answers were evaluated by presenting pictures of these items, one at a time, for the child to name the item. Each target answer was probed once and was considered as a known tact if the child named it accurately. Additionally, the FFC of each target answer was evaluated in the form of selection-based responses presented in a field of three pictures. For example, to test the FFC for the target answer “strawberries,” a picture of a strawberry and two pictures of other items were presented with the verbal antecedents, “Point to the thing that you can eat,” “Which one has small dots?” and “Show me a fruit.” The criterion for each target answer’s FFC was 100% accuracy for each trial. A correct response was reinforced with praise while incorrect responses were ignored in the probe trials.

After the assessment, each child’s unknown tacts were trained to criterion using the echoic-to-tact procedure described by Greer and Ross (2008); the unknown FFCs were trained with the selection-based trials using gestural prompts. Each pre-experimental training session contained 10 tact trials for unknown tacts and 10 selection trials for unknown FFCs. The training sessions continued until all tacts and FFCs met the mastery criteria of 100% accuracy in probe trials. Dede had 7 target answers with unknown FFCs for Question 1, 10 for Question 2, and 4 for Question 3. Tian had 9, 14, and 9 target answers with unknown FFCs for Questions 1, 2, and 3, respectively. We implemented pre-experimental training for these unknown tacts and FFCs three to four sessions per week. It required 2 weeks for Dede and 4 weeks for Tian to complete pre-experimental training.

#### Probe Trials Across Conditions

The probe trials were conducted in the following manner: the instructor first obtained the child’s attention, delivered the target question (e.g., “Name some red things, as many as you can”), and waited 3 s for the child to respond. Next, the instructor listened to the child’s responses until the child paused for 3 s. The instructor then asked, “Are you done?” to ensure the child had no more answers. If the child added more varied answers, these were recorded as correct answers, and the instructor provided reinforcement to end the trial. Conversely, if the child said “no more” or did not provide additional answers within 3 s, the instructor provided reinforcement for correct responses and concluded the trial. A reinforcer was delivered in a VR2 schedule (e.g., “Yeah, red apples, strawberries, stop signs, and red grapes are red things,” and delivering one or two reinforcers) at the end of each probe trial. Incorrect responses were ignored.

#### Training

Two female graduate students in special education served as instructors in this study. Each training session consisted of five training trials. The instructor selected five target answers for each training trial. One training trial consisted of (a) the instructor asking the target question, (b) a 3 s time delay, (c) the child’s response(s), (d) praise for each response and ignoring for incorrect responses, if any, (e) an intraverbal prompt for a target answer, and (f) praise for a correct prompted answer and an echoic prompt for an incorrect response. Immediately following the consequence delivered for a target answer, steps (e) and (f) were repeated for the other four target answers. That is, the instructor moved on to the next intraverbal prompt for another target answer. The five target answers and their FFC were presented in a random order for each training trial. If the child independently responded with a target answer after the question was asked, the prompt for that target answer was omitted from that training trial. If a target answer reached criterion, another new target answer was added to the next training trial. Consistent with probe trials, tangible reinforcers identified in the preference assessment were delivered in a VR2 schedule for independent/unprompted correct responses in training trials. A training trial was concluded when all five target answers were presented. A preferred activity or a snack break was provided following each trial. A training session ended when five training trials were complete. Each session lasted approximately 20 to 30 min. Each child received four to five training sessions per week. The training condition for all three target questions took approximately 6 weeks for Dede and 8 weeks for Tian. The training condition for the target questions ended when the child achieved criterion performance for all target answers listed in Tables 1, 2 and 3.

An example of a training trial is as follows: After obtaining the child’s attention, the instructor delivered the target question (e.g., “Name some red things, as many as you can”) and waited 3 s for the child to respond. If there was no response within 3 s, the instructor provided an intraverbal prompt of a target answer to prompt for a correct answer (e.g., “Guess what? This red thing is a fruit, you can eat it, and it has dots on it and green leaves on top.”). The instructor then reinforced the child’s correct answer or gave the answer to the child (e.g., “This red thing is a strawberry. Say ‘A strawberry’”), if the child did not provide the correct answer after the prompt. If child provided a response (e.g., “a cherry”) that was incorrect to the intraverbal prompt (e.g., described a strawberry) but correct to the initial question (e.g., “Tell me some red things”), the response was not considered as a correct response. The instructor proceeded with the error correction, “The red thing I just described was a strawberry.” (This type of response did not occur with the two children involved in this study). The instructor then continued to provide another FFC prompts for the subsequent target answer until all five target answers were presented to end a training trial.

**Fig. 1:**
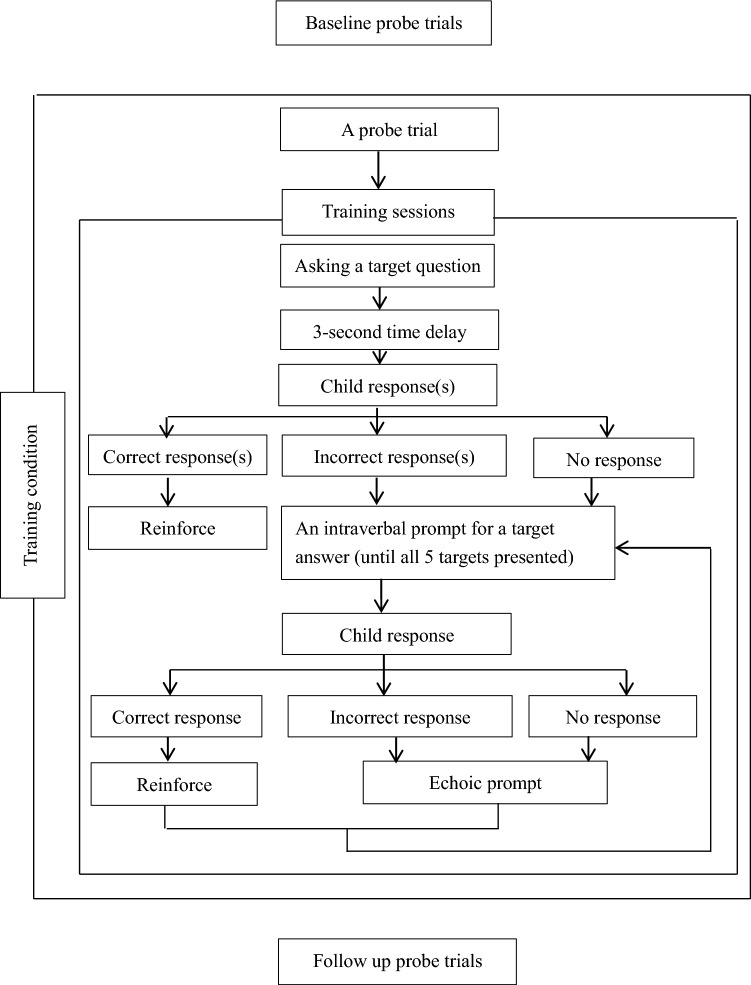
Sequence of the baseline, training, follow-up conditions, and procedures of a training trial

In the same example, if the child provided an answer or several answers (e.g., “chili peppers, red apples, and red grapes) following the target question, the instructor waited for the child to finish all answers and provided reinforcement for each correct answer while ignoring incorrect answers, if any. The instructor then provided intraverbal prompts for other target answers, one at a time, until all target answers for that training session were presented to complete the training trial. Figure 1 presents the sequence of the baseline, training, follow-up conditions, and the procedures used in a training trial.

### Procedural Integrity and Interobserver Agreement

All probe trials and training sessions were videotaped. Two graduate students of special education were trained to assess procedural integrity and interobserver agreement (IOA) by watching the recorded sessions. The assessors independently checked the accuracy of each implementation step in the probe trials and training trials described in the procedure section, including the delivery of the antecedents and the consequences based on each child’s response(s). The assessors also recorded the child’s responses to obtain point-to-point IOA data. Procedural integrity was assessed in at least 30% of the training sessions across the three target questions for each child and in at least 30% of probe trials across the baseline, training, and follow-up conditions for each child. The percentage of procedural integrity was calculated using this formula: accurate steps of implementation ÷ total steps of implementation × 100. The integrity was 100% for the training sessions and 100% for the probe trials for the sessions observed.

Point-to-point IOA was assessed in at least 30% of the probe trials across all conditions for each child. The formula for point-to-point IOA was: number of agreement ÷ total number of agreement and disagreement × 100. Point-to-point IOA was 100% for all sessions observed.

### Social Validity

To assess social validity, the instructors conducted a paper-based survey (Appendix 1) with parents and headteachers following the completion of the training. The survey consisted of 10 items, including training acceptability (Items 1–3), feasibility (Items 4–5), satisfaction (Items 6–9), and an open-ended question for suggestions. Each item was rated on a 5-point Likert scale (1 = strongly dissatisfied or disagree to 5 = strongly satisfied or agree).

## Results

Table 4 displays the mean and standard deviation for the number of prompts provided, new target answers added, and target answers mastered per training session for both children. Both children required over 20 prompts per session in the beginning but decreased progressively to fewer than 10 prompts per session toward the end of the training condition. Overall, the number of target answers mastered and new targets added were stable across the training sessions.

**Table 4:** The mean frequency and SD for prompts provided, new targets answers added, and target answers mastered for target questions per training session for both children

	Dede			Tian		
	*M*	*SD*	Range	*M*	*SD*	Range
Q1						
Prompts	16.08	7.14	2–15	18.65	5.75	5–28
New targets added	1.75	1.92	0–5	1.0	1.31	0–5
Target mastered	1.91	1.73	0–5	1.0	1.0	0–4
Q2						
Prompts	14.58	5.81	4–25	12.45	4.57	2–20
New targets added	1.67	1.72	0–5	1.1	1.37	0–5
Target mastered	1.75	1.48	0–4	1.25	1.11	0–4
Q3						
Prompts	14.33	5.93	5–25	9.0	6.38	2–22
New targets	1.67	2.06	0–5	1.67	1.73	0–5
Target mastered	1.67	1.73	0–5	1.67	1.41	0–4

### Divergent and Novel Responses to Target Questions

**Fig. 2:**
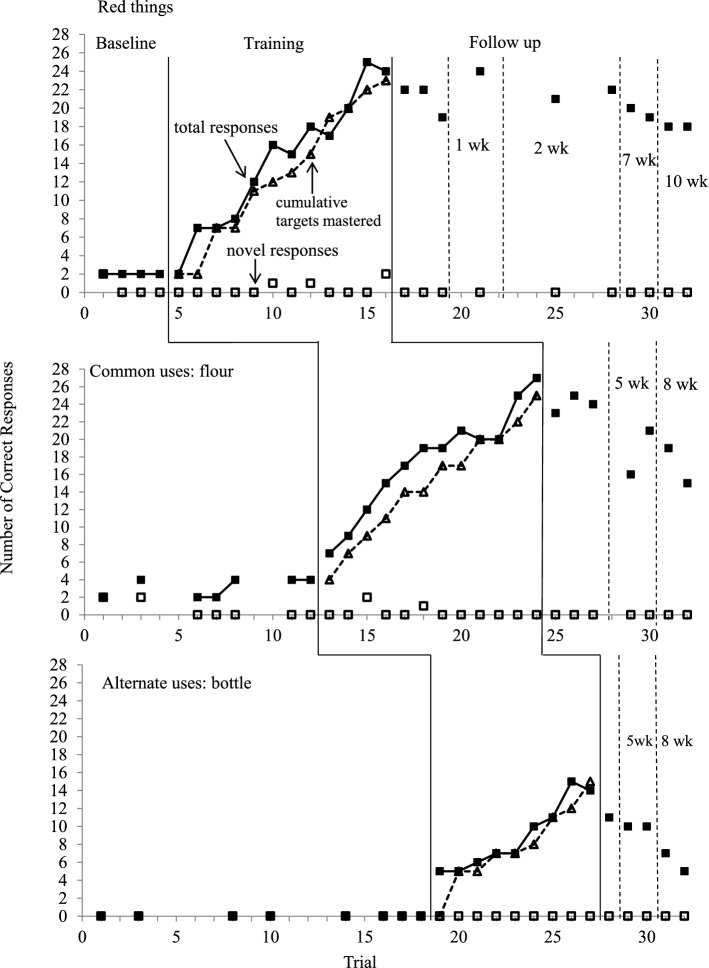
Number of diverse and novel responses in probe trials and the cumulative number of target answers mastered for target questions across all conditions for Dede

**Fig. 3:**
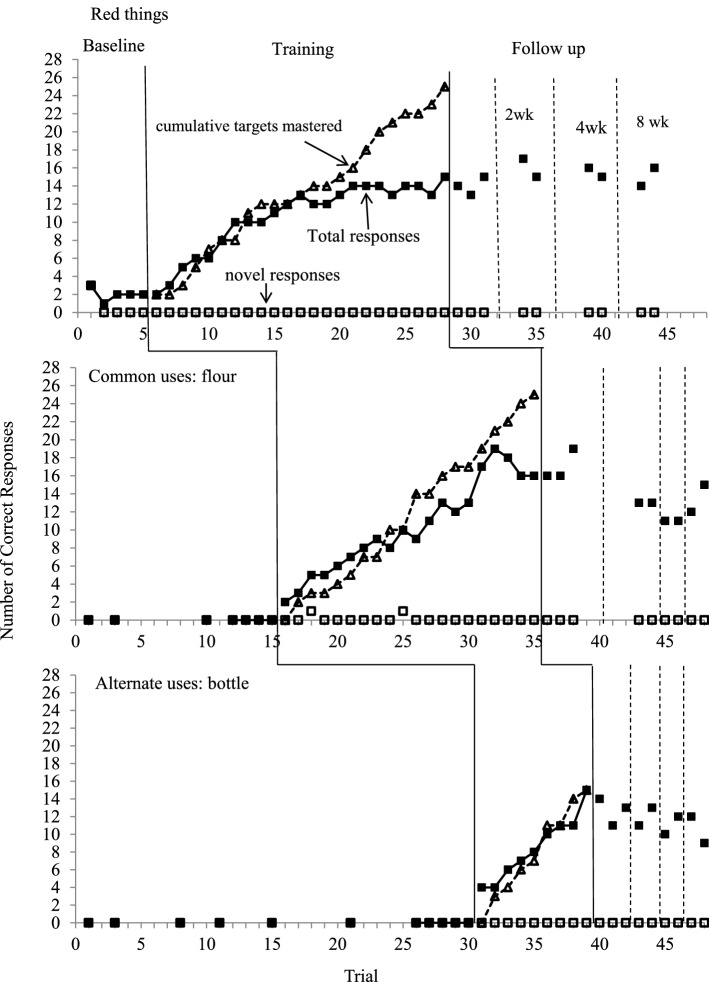
Number of diverse and novel responses in probe trials and the cumulative number of target answers mastered for target questions across all conditions for Tian

Figures 2 and 3 depict the number of divergent responses and novel responses in probe trials, and the cumulative number of target answers mastered for three target questions across conditions for Dede and Tian, respectively.

#### Dede

Compared to the number of divergent responses at baseline, Dede’s responses for all three target questions started from a low level but gradually increased to a high level in the training condition and maintained acquired responses in the follow-up condition. No data point at follow-up overlapped with baseline values for all three target questions.

At baseline, Dede provided 2 responses per trial for Question 1, 2 to 4 responses for Question 2, and zero responses for Question 3. When intraverbal prompts were introduced, Dede’s responses increased from a low level and gradually ascended to a high level in 12 sessions for Question 1 (range 2–24) and Question 2 (range 7–27), and in 9 sessions for Question 3 (range 5–15). He also provided a total of 4 novel responses for Question 1, 3 for Question 2, and zero for Question 3.

The number of divergent responses for all target questions was maintained at a high level immediately following the completion of the training but decreased to a slightly lower level in the later follow-up condition. Dede maintained 60% to 76% (15–19 responses) of the taught target answers for Questions 1 and 2 in 10-week probe trials but decreased to 33% to 46% (5–7 responses) of the taught target answers for Question 3 in 8-week probe trials. He did not provide any novel responses at follow-up.

As shown in the cumulative number of target answers mastered for each session, the number of divergent responses increased along with the addition of new target answers in the training condition. The raw data indicated that Dede provided some varied responses and the responses were in a random order for each probe trial in the training condition.

#### Tian

Tian’s number of divergent responses for all three questions had a similar pattern as Dede. His correct responses gradually increased from a low level to a high level after the introduction of the training and maintained at a high level after the training. No data point at follow-up overlapped with baseline values for all three target questions.

At baseline, Tian provided 2 to 3 responses per trial for Question 1 and zero response for Questions 2 and 3. His responses started at a low level but gradually increased to a high level with a range of 2 to 15 for Question 1, 2 to 19 for Question 2, and 4 to 15 responses for Question 3 in the training condition. He required 23, 20, and 9 training sessions to achieve criterion performance for Question 1, Question 2, and Question 3, respectively. He had a total of 2 novel responses for Question 2 but did not have any for Questions 1 and 3.

Tian’s number of responses for all target questions increased along with the mastery of more target answers in the training condition. An examination of the raw data indicated that he provided an invariant pattern of responses in the beginning sessions when the number of acquired target answers remained low. His responses for all target questions appeared in a random order in the training condition as the number of mastered target answers increased.

Tian maintained the acquired responses for Question 1 at a high level in the follow-up condition. The number of responses for Questions 2 and 3 was maintained at a high level immediately following the completion of the training but decreased to a slightly lower level at later follow-up. Tian maintained 48% to 64% (12–16 responses) of the taught target answers for Questions 1 and 2 and 60% to 80% (9–12 responses) of the taught target answers for Question 3 in 8-week probe trials. No novel responses occurred at follow-up.

### Generalization to Similar Questions

**Fig. 4:**
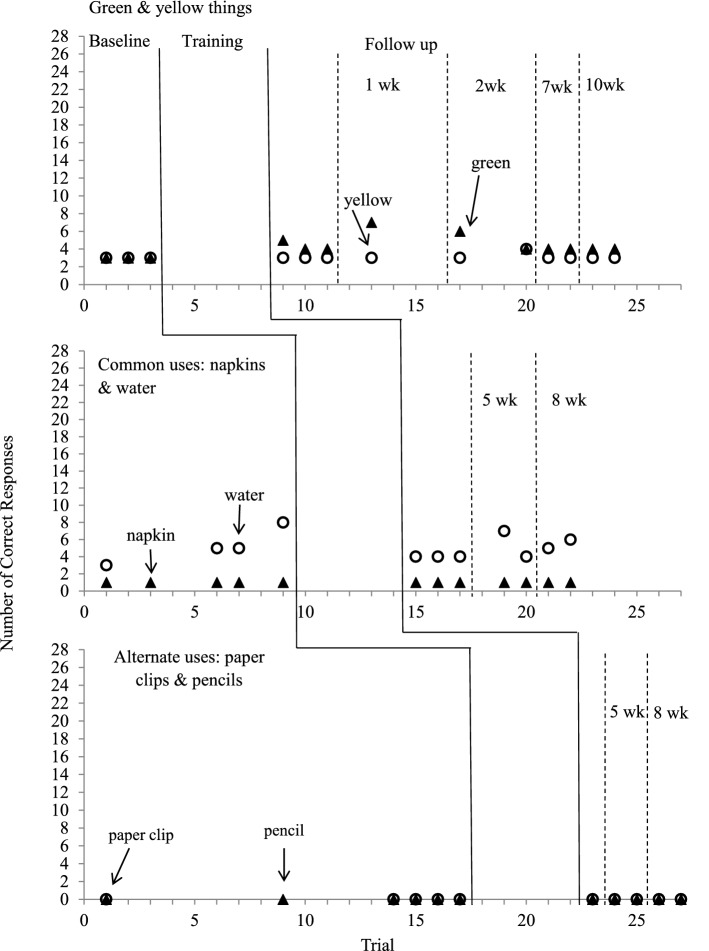
Number of correct responses for generalization questions in baseline and follow-up conditions for Dede

**Fig. 5:**
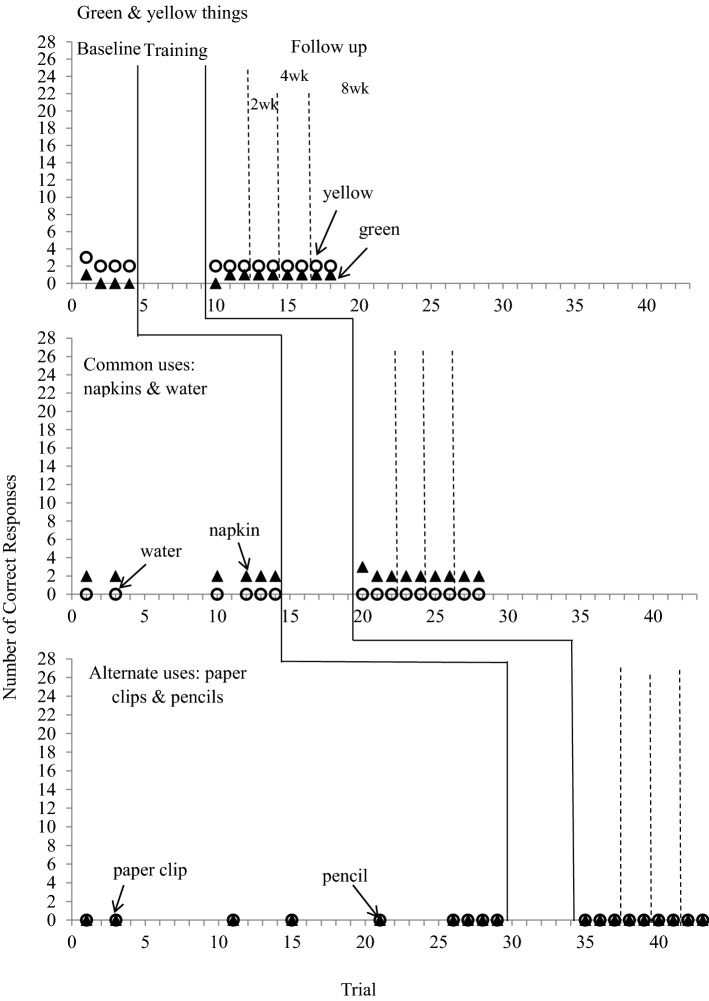
Number of correct responses for generalization questions in baseline and follow-up conditions for Tian

Figures 4 and 5 depict the number of correct responses to generalization questions before and after the training for both children.

#### Dede

Dede provided 3 correct responses for green things and for yellow things at baseline. The number of responses increased slightly after the mastery of Question 1 (green things range 4–7; yellow things, range 3–4 responses). For common uses, he provided 3 to 8 responses for water and one response for napkins at baseline and the responses remained at the same level after mastering Question 2 (water range 4–7; napkin range 1–1). He did not provide any response for untaught alternate uses questions before and after the training for Question 3.

#### Tian

Tian’s correct responses for generalization questions at baseline did not differ from those at follow-up. His responses for generalization questions were at the same level before and after acquiring Question 1 (green things, range 0–1 at baseline, 0–1 at follow-up; yellow things, range: 2–3 at baseline, 2–2 at follow-up) and Question 2 (napkin, range 2–2 at baseline; 2–3 at follow-up; water, 0 at baseline and follow-up). He did not provide any response for generalization questions about alternate uses in baseline and follow-up conditions.

### Social Validity

The parents and headteachers responded to the social validity survey and the average ratings were 4.78 (*SD* = .45) on training acceptability, 4.59 (*SD* = .58) on feasibility, and 4.89 (*SD* = .56) on the satisfaction of the training. The parents and teachers reported positive changes in Dede’s and Tian’s verbal responses when interacting with them. Dede’s teacher reported that Dede used to have no response when she asked him questions but this was changed after the training. For example, when she asked, “Who are your teachers?” Dede provided all four teachers’ names in his classroom. Tian’s mother said that Tian started to try different foods and did not always say that he only liked gummies and skittles when asked, “What foods do you like to eat?” The teachers indicated that they have successfully used the same teaching strategy (i.e., providing the FFC of an item) to increase student responses for all children in their classrooms. For example, Tian’s teacher reported that she used this strategy to provide hints for the question, “What places have you visited?” and Tian was able to name more than five places. The parents also reported that they have enjoyed using this teaching strategy to solicit multiple responses in conversation with their children. Dede’s mother reported that she used this strategy as if they were playing a “guessing” game, and they both enjoyed it. She commented that it was “amazing” to see Dede talking about different types of toys and play activities that he was not interested in earlier and attributed this change to the training. Tian’s mother felt that this strategy was easy to implement and would like to try it for bedtime reading.

## Discussion

The study evaluated the effects of the FFC intraverbal prompts on the acquisition and generalization of divergent and novel responses to intraverbals requiring multiple responses for two children with ASD. The intraverbal prompts were effective in increasing the number of divergent responses to three target questions for both children. The children maintained acquired responses to the questions for 8 weeks after the completion of the training. Novel responses emerged at a low level, suggesting that novel responses in intraverbals remained challenging for the children with ASD. Generalization to similar questions did not occur.

### Divergent Responses to Target Questions

Consistent with previous research (Feng et al. 2017; Grannan and Rehfeldt 2012; Lee et al. 2017), the results of this study indicated that children with ASD acquired complex intraverbal behavior involving both convergent and divergent control. The FFC intraverbal prompts effectively increased the number of divergent responses to all three questions after the completion of the training. For each target question, the number of divergent responses started at a low level, gradually ascended to a high level in the training condition, and was maintained in the follow-up condition. As the number of divergent responses for Question 1 increased in the training condition, responses to subsequent target questions at baseline remained at a low level. The comparison of baseline-training data between and within each target question indicated that divergent control to intraverbals was established through the FFC intraverbal prompts involving convergent control or conditional discriminations. It is important to note that prompts (e.g., “What else?”) were not provided between responses, and the number of responses was not specified in the questions. Restrictively speaking, divergent control was not established if a verbal antecedent stimulus was needed for one response at a time. Specifying the number of responses may potentially create a ceiling or rote responding pattern (Lee et al. 2017). Instead, the child was simply asked to provide as many responses as possible.

Examinations of each child’s individual responses showed that a rote pattern of responding (e.g., the same objects in the same order) occurred in the beginning sessions for Tian. However, such a pattern did not continue with the addition of target answers, indicating the reduction or elimination of undesired rote responding with the training. We did not implement additional procedures to interrupt rote responses as described in Feng et al. (2017). For example, when a rote pattern of responding was observed, the instructor immediately interrupted by repeating the child’s rote response(s) to prevent the child from emitting the same response(s) each time (e.g., “The red things are strawberries, and what else?”). It is possible that the picture prompts used in Feng et al. (2017) directly evoked a tact response through which a child did not have to attend to the question asked. In this study, the intraverbal prompts had no point-to-point correspondence and required the child to conditionally discriminate three verbal stimuli (i.e., FFC of a target answer) in order to emit a correct response, thereby reducing the probability of rote responding. Additionally, the target questions included in the present study were selected from different categories, as opposed to only one category (e.g., fruits of different colors) in Feng et al. (2017). Therefore, functional intraverbal responses are more likely to be established and strengthened through intraverbal prompts with questions from different categories.

Both children maintained a relatively greater number of responses per trial for Questions 1 and 2, compared to that of Question 3. One explanation was that Questions 1 and 2 had 25 target answers while Question 3 had 15 target answers. Dede maintained the percentage of taught target answers for Questions 1 and 2 at a similar level, but Question 3 was maintained at a relatively low level in the 8-week follow-up probe trials. Tian maintained a similar percentage of taught target answers for all questions in the 8-week probe trials. One plausible explanation for Dede’s stronger maintenance results for Questions 1 and 2 was that he received a relatively greater number of training trials for Questions 1 and 2, compared to the number of training trials received for Question 3.

### Novel Responses to Target Questions

Novel responses occurred in both children but were limited to red things and common uses for flour, not creative uses for bottles. Consistent with previous research (Feng et al. 2017; Lee et al. 2017, 2019), the number of novel responses occurred, but they were at a relatively low level, suggesting that response novelty remains a challenge for children with ASD. Therefore, it is necessary to develop interventions to target response novelty specifically. The low number of novel responses in this study was partially explained by the high number of target responses for each question (e.g., 25 target responses for Questions 1 and 2, 15 for Question 3) during training which made novel responses less likely to occur during the later stage of the training.

The absence of novel responses to the question of alternative uses was partly explained by the complexity involved in the question, as it required children to create novel responses beyond their daily experiences. It is possible that this particular question requires a certain “imagination” outside the realm of reality, which can be challenging for children with ASD (Craig and Baron-Cohen 1999). As previous research has indicated that typically developing children’s creative responses in various contexts can be improved through instructions, reinforcement, and practice opportunities (Glover and Gary 1976; Goetz and Baer 1973), interventions for improving creativity beyond ordinary experiences can include motivational arrangements to make connections between reality and imagination in various contexts, such as play, problem solving, and conversations. Additionally, a procedural refinement is to insert a delay prompting procedure into intraverbal prompts by stating one feature, waiting for 3 s, and stating the next. Stating one FFC at a time provides a broader range of potential items and thus may lead to diverse and novel responses. Whether such a procedure would result in improved acquisition of creative responses warrants further investigations.

### Generalization to Similar Questions

Generalization to similar questions has not been evaluated in previous studies of intraverbal responses involving both convergent and divergent control (Feng et al. 2017; Grannan and Rehfeldt 2012; Lee et al. 2017). In this study, generalized responses to similar questions did not occur for both children after the acquisition of target questions. In Feng et al. (2017), generalization for similar categorical questions started to emerge after the participant had acquired at least three similar categorical questions. This observation suggests that teaching one question in each category was not sufficient for generalization to occur. Therefore, explicit instruction on several similar questions in the same category is necessary to promote generalization. Additionally, only the tacts and the FFC for the answers of the target questions were assessed, not those for the generalization questions. The poor performance for the generalization questions was potentially due to the lack of tacts and selection responses of the FFC for the answers related to generalization questions.

### Strengths, Limitations, and Future Directions

The results of this study demonstrated a functional relationship between the intraverbal prompts and the increased number of divergent responses across three target questions requiring multiple responses. As part of the experimental control which was to isolate the effects of intraverbal prompts on independent responses, the reinforcement was held constant in probe sessions across conditions. Including reinforcement for correct responses at baseline could rule out the possibility that reinforcement alone is sufficient to increase the number of intraverbal responses. Additionally, the use of FFC intraverbal prompts to establish functional intraverbals can potentially eliminate prompt dependency and increase convergent control to multiple verbal antecedent stimuli, compared to picture or echoic prompts. The intraverbal prompts can be incorporated into any instruction and are relatively easy to implement. The results of social validity also supported the feasibility of this intervention as teachers and parents used this approach in their instructions or interactions with children.

However, the use of intraverbal prompts by teachers and parents during the course of the study could possibly influence the results of the study. Although they used the strategy for other activities not related to the target questions, additional training outside of the study posed an extraneous variable interfering with the interpretation of the data. It is necessary for future researchers to consider the influence of additional training received by children in school or at home.

As discussed, future researchers may consider teaching multiple similar questions before assessing generalized responses to untaught questions in the same category as training one question may not provide sufficient multiple-exemplar experiences for generalization to occur. Another limitation was the lack of assessment on the tacts and selection responses of the FFC for the items included in generalization questions. It is necessary to include an assessment of potential items and their FFC for generalization questions in future studies.

The results of Grannan and Rehfeldt (2012) indicated that divergent responses to categorical questions emerged without explicit instruction after a sequenced instruction of relevant skills (i.e., simple tact, category tact, and matching) were established. Future researchers can adapt a similar procedure to increase the number of divergent responses to intraverbals and examine its effects on acquisition and generalization to similar questions. Additionally, it is necessary to develop and evaluate interventions aimed at establishing multiple control in intraverbal relations that will teach children with ASD the effective use of intraverbals in potentially more creative contexts, such as play activities, book reading, and social conversation about imagination. More research is needed in this important area.

### Implications

The results of this study have important implications for educators and practitioners working with children with ASD in applied settings. It is important to establish convergent and divergent control when teaching complex intraverbal behavior, such as responding to complex questions with multiple answers. Increasing multiple control in intraverbal behavior is necessary to establish and strengthen functional intraverbal repertoire for children with ASD who engage in invariant response patterns. Establishing divergent control by teaching them to provide multiple responses to a single question is one of the initial steps to facilitate creative responses. The use of FFC intraverbal prompts can be incorporated into interaction and conversation in various contexts to strengthen complex intraverbal behavior in children with ASD.

## Appendix 1: The Social Validity Questionnaire

1: Strongly disagree/dissatisfied; 2: Disagree/dissatisfied; 3: Neutral/no opinion; 4: Agree/satisfied, 5: Strongly agree/satisfied

**Table:**

	Item\rating	1	2	3	4	5
1.	The content and the teaching format are appropriate					
2.	The training is important to the child					
3.	The training meets the child’s learning needs.					
4.	The time/duration for training is arranged properly.					
5.	The location of the training is appropriate.					
6.	The training is effective.					
7.	Are you satisfied with the overall progress of the child?					
8.	Are you satisfied with the results of the training?					
9.	Will you recommend this training to other parents?					

10. Please tell us your comments and/or your suggestions to improve the training
